# Evaluation of Combined Chemotherapy and Genomic-Driven Targeted Therapy in Patient-Derived Xenografts Identifies New Therapeutic Approaches in Squamous Non-Small-Cell Lung Cancer Patients

**DOI:** 10.3390/cancers16162785

**Published:** 2024-08-07

**Authors:** Didier Decaudin, Fariba Némati, Julien Masliah Planchon, Agathe Seguin-Givelet, Marine Lefevre, Vesnie Etienne, Harry Ahnine, Quentin Peretti, Laura Sourd, Rania El-Botty, Lea Huguet, Sarah Lagha, Nadia Hegarat, Sergio Roman-Roman, Ivan Bièche, Nicolas Girard, Elodie Montaudon

**Affiliations:** 1 Laboratory of Preclinical Investigation, Department of Translational Research, Institut Curie, PSL University Paris, 75005 Paris, France; fariba.nemati@curie.fr (F.N.); vesnie.etienne@curie.fr (V.E.); laura.sourd@curie.fr (L.S.); rania.el-botty@curie.fr (R.E.-B.); lea.huguet@curie.fr (L.H.); 2 Department of Medical Oncology, Institut Curie, 75005 Paris, France; sarah.lagha@curie.fr (S.L.); nadia.hegarat@curie.fr (N.H.); nicolas.girard2@curie.fr (N.G.); 3 Department of Genetic, Institut Curie, 75005 Paris, France; julien.masliahplanchon@curie.fr (J.M.P.); ivan.bieche@curie.fr (I.B.); 4 Department of Thoracic Surgery, Curie-Montsouris Thorax Institute, Institut Mutualiste Montsouris, 75014 Paris, France; agathe.seguin-givelet@imm.fr; 5 Faculty of Medicine SMBH, Paris 13 University, Sorbonne Paris Cité, 75013 Bobigny, France; 6 Department of Pathology, Institut Mutualiste Montsouris, 75014 Paris, France; marine.lefevre@imm.fr; 7 Department of Translationnal Research, Institut Curie, PSL University Paris, 75006 Paris, France; sergio.roman-roman@curie.fr; 8 Paris Saclay University, University of Versailles Saint-Quentin-en-Yvelines (UVSQ), 91405 Versailles, France

**Keywords:** squamous NSCLC, PDXs, NGS-defined targeted therapy, chemotherapy, combination

## Abstract

**Simple Summary:**

Non-small-cell lung cancer is characterized by high morbidity and mortality. Currently, the precision medicine approach in the adenocarcinoma subtype of non-small-cell lung cancer aims to identify genomic alterations that can be targeted in individual patients and offer them appropriate treatment. Several biomarker-guided therapies have been approved, targeting genes frequently altered in adenocarcinoma, such as *EGFR*, *BRAF*, *MET*, *ALK*, *ROS1*, *RET* and *NTRK*. To overcome the emergence of resistance and increase the efficacy of these targeted therapies, the combination of chemotherapy and targeted therapy has been studied and validated in adenocarcinoma patients with *EGFR* mutations. This study proposes to examine whether this type of combined approach, which is difficult to study in clinical trials, could be more widely used by targeting other genetic alterations in the MAPK/PI3K pathways or against alterations in the *CDNK2A* gene in adenocarcinomas but also in squamous-cell carcinomas.

**Abstract:**

The combination of chemotherapy and targeted therapy has been validated in non-small-cell lung cancer (NSCLC) patients with *EGFR* mutations. We therefore investigated whether this type of combined approach could be more widely used by targeting other genetic alterations present in NSCLC. PDXs were generated from patients with NSCLC adenocarcinomas (ADCs) and squamous-cell carcinomas (SCCs). Targeted NGS analyses identified various molecular abnormalities in the MAPK and PI3K pathways and in the cell cycle process in our PDX panel. The antitumor efficacy of targeted therapies alone or in combination with chemotherapy was then tested in vivo. We observed that trametinib, BKM120, AZD2014 and palbociclib increased the efficacy of each chemotherapy in SCC PDXs, in contrast to a non-insignificant or slight improvement in ADCs. Furthermore, we observed high efficacy of trametinib in *KRAS*-, *HRAS*- and *NRAS*-mutated tumors (ADCs and SCCs), suggesting that the MEK inhibitor may be useful in a wider population of NSCLC patients, not just those with *KRAS*-mutated ADCs. Our results suggest that the detection of pathogenic variants by NGS should be performed in all NSCLCs, and particularly in SCCs, to offer patients a more effective combination of chemotherapy and targeted therapy.

## 1. Introduction

Non-small-cell lung cancer (NSCLC) is the leading cause of cancer-related mortality worldwide [1]. NSCLC is divided into three major histological subtypes, from the most common to the least common: adenocarcinoma (ADC), squamous-cell carcinoma (SCC), and large-cell carcinoma. Most cases are diagnosed at an advanced or metastatic stage. Chemotherapy has been the mainstay of treatment for NSCLC patients for around three decades. Standard chemotherapy regimens include platinum-based therapy combined with an antimetabolic agent (cisplatin plus pemetrexed) for ADCs or a fusiform poison (carboplatin plus paclitaxel) for SCCs. Over the past decade, the sequencing of the human genome has enabled the emergence of new biological therapies for NSCLC, targeting specific molecular alterations known as driver mutations. In the clinic, patient treatment decisions are based on observed oncogenic molecular alterations [2], as these enable the administration of targeted agents that are more effective than traditional chemotherapy regimens [3,4]. This is particularly true for non-squamous NSCLC, where most of these alterations are observed. However, SCCs also display various oncogenic alterations that could guide these precision medicine approaches [5].

Recently, combination of EGFR tyrosine kinase inhibitors (TKI) with platinum-based chemotherapy demonstrated a significant survival benefit when compared to targeted agents alone in ADCs [6,7]. These data suggest such combinations may change the clonal evolution of NSCLC tumors, through the eradication of TKI-resistant clones leading to acquired resistance and disease progression. 

A key question in the clinic is whether such combination approaches are relevant in other oncogenic alterations both in non-squamous and squamous NSCLCs. It is now well demonstrated that patient-derived xenografts (PDXs) constitute the best models for preclinical studies addressing these questions [8]. Indeed, the stability of the genomic and gene expression profiles of PDXs in mice at first transplantation and during the in vivo maintenance of the model is a key relevant characteristic of these models [9]. Furthermore, it has recently been shown that PDXs can be highly predictive models for therapeutic response in cancer patients [10]. 

We therefore investigated whether the combination of standard chemotherapy and targeted therapy in NSCLC PDXs, with both treatments defined according to histopathological subtypes and tumor molecular abnormalities, could improve in vivo antitumor responses.

## 2. Materials and Methods

### 2.1. Patients and Tumor Samples

The PDX bank was established from engraftment of fresh human tumors coming from surgically resected patients with confirmed NSCLC. The project was hosted under the institutional tissue collection umbrella protocol of Institut Mutualiste Montsouris dedicated to thoracic malignancies (EUdract 2017-A03081-52 approved by the Ethics Committee CPP SUD-EST I on 15 December 2017). Consent was obtained for all patients. The selection of patient was based on several criteria: confirmed diagnosis of NSCLC; all histological subtypes (ADC, large-cell neuroendocrine carcinoma, SCC); and chemo- and immune-naive operable patients at the Institut Mutualiste Montsouris (Thoracic Departement, Paris, France) (TNM staging 8th edition) (except for the patient at the origin of LCIM28 who received preoperative immunotherapy). For each patient, the following clinical data were collected: age, sex, smoking habits, tumor histology, and stage of disease. In total, 31 tumors from confirmed NSCLC diagnoses were implanted into the fat pads of Swiss nude mice, as previously described [11].

### 2.2. Animals and Animal Facilities

Mice were purchased from Charles River Laboratories and maintained under specific pathogen-free conditions. The care and animal housing were in accordance with institutional guidelines and with the recommendations of the French Ethics Committee (authorization APAFiS No. 25870-2020060410487032-v1 given by the national authority). The housing facility was kept at 22 °C (±2 °C) with a relative humidity of 30–70%. The light/dark cycle was 12 h light/12 h dark. 

### 2.3. Histopathological Analyses

Formalin-fixed, paraffin-embedded sections were de-paraffinized in xylene and rehydrated in a gradient of ethanol prior to processing. Hematoxylin, eosin, and saffron staining were carried. Histological examination was realized on each PDX and compared with the histologic features of the corresponding patient’s tumor.

### 2.4. Somatic Genetic and Genomic Alteration Analyses

PDX genomes were analyzed by targeted next-generation sequencing (NGS). A custom NGS panel called DRAGON (Detection of Relevant Alterations in Genes involved in Oncogenetics by NGS) and marketed by Agilent under the name of SureSelect CD Curie CGP has been recently developed in the genetics department, specifically for the molecular analysis of tumors for single nucleotide variant, copy-number variation (CNV), microsatellite instability, and tumor mutational burden status. It is composed of 571 genes of interest in oncology from diagnosis, prognosis, and theranostics, including potential therapeutic targets (Supplemental Table S1). All studied genetic abnormalities and modalities of analyses are described in Passeri et al. [12].

### 2.5. In Vivo Efficacy Studies

Firstly, the sensitivity of PDXs to standard chemotherapy combinations was evaluated in 15 models. Based on histological characteristics, mice received pemetrexed (100 mg/kg, 1x/w, IP) plus cisplatin (4 mg/kg (1x/3w, IP) for ADC, carboplatin (66 mg/kg, 1x/3w, IP) plus paclitaxel (20 mg/kg, 1x/3w, IP) for SCC, etoposide (10 mg/kg, 3x/3w, IP) plus cisplatin (4 mg/kg, 1x/3w, IP) for large-cell neuroendocrine carcinoma, and gemcitabine (100 mg/kg, 1x/w, IP) plus cisplatin (1 mg/kg, 1x/w, IP) for sarcomatoid carcinoma.

Secondly, thirteen NSCLC PDXs (out of 16 established PDX models) in which an NGS study (panel “DRAGON”) was performed were selected for in vivo experiments, in order to assess the efficacy of targeted therapies alone and in combination with chemotherapies. Afatinib (EGFR inhibitor), trametinib (MEK inhibitor), buparlisib (BKM120) (PI3K inhibitor), vistusertib (AZD2014) (mTORC1/2 inhibitor), palbociclib (CDK4/6 inhibitor), birabresib (BET bromodomain inhibitor), and tazemetostat (EZH2 inhibitor) were purchased from MedChemExpress. Afatinib, trametinib, BKM120, AZD2014, palbociclib, birabresib, and tazemetostat were administrated orally 5 days per week at a daily dosage of 15 mg/kg, 0.4 mg/kg, 15 mg/kg, 10 mg/kg, 50 mg/kg, 30 mg/kg (twice-a-day), and 150 mg/kg, respectively. 

In in vivo experiments, the procedure of tumor graft, as well as tumor growth assessment, evaluation of the efficacy of tested therapies, and time sacrifice were performed, as previously reported [13]. The quality of the antitumor response was determined as follows: A decrease in tumor volume of at least 50% was classified as huge or complete response, a volume change between −50% and +35% was considered as stable disease, and an increase in tumor volume of at least 35% was identified as partial response (TGI: percentage of tumor growth inhibition > 50%) or progressive disease (TGI < 50%) (Supplemental Figure S1) [14]. To assess treatment response in relation to individual mouse variability, we determined the overall response rate (ORR) for each treated mouse. For each PDX model, the ORR was calculated for each treated mouse as follows: ORR= [(RTVt/mRTVc)]-1, where RTVt is the relative tumor volume of the treated mouse and mRTVc is the median relative tumor volume of the corresponding control group at the end of treatment.

Finally, to study the impact of treatments on the time to tumor growth delay, we also calculated the probability of progression (doubling time and time to reach RTV = 4), as previously described [13]. Statistical analysis of tumor growth was performed using the unpaired Kruskal–Wallis test with Dunn’s multiple comparison test or the unpaired Mann–Whitney test for comparisons between two groups. Statistical analysis of the progression curves was performed using the log-rank test.

## 3. Results

### 3.1. Development of a Representative Panel of NSCLC PDXs and Identification of Targeted Genomic Alterations

A panel of 16 NSCLC PDXs, representative of the different histological subtypes of the disease, was obtained from the implantation of 31 primary tumor samples in nude mice (uptake rate: 51.6% and average tumor uptake time = 133 days). This panel includes eight ADCs (50%), five squamous NSCLCs (31%), two large cell carcinomas (13%), and one sarcomatoid sub-type (6%) (Supplemental Figure S2a). No significant correlation between in vivo tumor uptake and the clinical and molecular characteristics of NSCLC patients (cumulative survival, age, gender, smoking habits, TNM stage, and histology) was observed (Supplemental Table S2). Interestingly, a significant correlation between in vivo tumor uptake and PDL1 expression in patients’ tumor cells (*p* = 0.02) was observed (Supplemental Figure S2c).

All the NSCLC PDXs were molecularly characterized at the genomic level by a targeted NGS analysis of 571 genes (the most frequently mutated genes in cancer) (Supplemental Table S1). Significantly mutated genes and CNV in putative cancer driver genes observed in our PDXs are shown in Supplemental Table S3. Of the 16 PDX models, 50% of the panel displayed pathogenic genomic alterations affecting the MAPK pathway (mutations of *KRAS* (25%), *NRAS*, *HRAS*, *RASA1*, *MAP2K4*, and *NF1*), 38% affecting the PI3K pathway (mutation or amplification of *PIK3CA*, mutations of *PIK3R1*, *PTEN* and *STK11*), and 38% of the panel presented mutations or homologous deletion of *CDKN2A/2B*. Only one PDX (ADC LCIM21) displayed an *EGFR* mutation (L858R exon21), representing 6% of the panel, as compared to 10% of *EGFR* mutations found in NSCLC patients. 

### 3.2. Low Sensitivity of Our PDX Panel to Standard Chemotherapies

**Figure 1:**
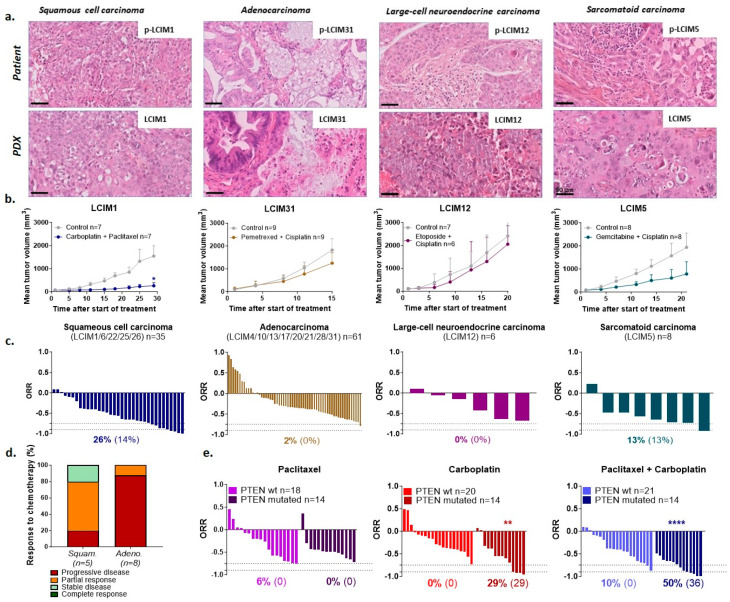
In vivo sensitivity of PDX NSCLC panel to standard chemotherapies. (**a**) Histological comparison with hematoxylin, eosin, and saffron staining between primary patients’ tumors (p-LCIM) and their corresponding xenografts (LCIM) (magnification ×400). (**b**) In vivo efficacy of carboplatin (66 mg/kg, 1x/3w, ip) plus paclitaxel (20 mg/kg, 1x/3w, ip) in squamous cell carcinomas, pemetrexed (100 mg/kg, 1x/w, ip) plus cisplatin (4 mg/kg, 1x/3w, ip) in adenocarcinomas, etoposide (10 mg/kg, 3x/3w, ip) plus cisplatin (4 mg/kg, 1x/3w, ip) in large-cell neuroendocrine carcinoma, and gemcitabine (100 mg/kg, 1x/w, ip) plus cisplatin (1 mg/kg, 1x/w, ip) in sarcomatoid carcinoma (RTV ± SEM). (**c**) Overall response rate (ORR): a percentage in bold corresponds to an ORR lower than −0.75 and a percentage in brackets corresponds to an ORR lower than −0.9. (**d**) Fraction of squamous carcinoma PDX panel and adenocarcinoma PDX panel responding to chemotherapy with progressive disease (TGI < 50% + % of change > 35%), partial response (TGI > 50% + % of change > 35%), stable disease (TGI > 50% + 35% > % of change > −50%), or complete response (TGI > 50% + % of change < −50%). (**e**) ORR to paclitaxel ± carboplatin in squamous carcinoma NSCLC PDX with or without *PTEN* mutation; a percentage in bold corresponds to an ORR lower than −0.75 and a percentage in brackets corresponds to an ORR lower than −0.9; statistical analysis of the efficacy of the treatment was performed by Wilcoxon test: * *PTEN*-mutated vs. *PTEN*-wt tumors; ** *p* < 0.01 and **** *p* < 0.0001.

Histological analyses between the primary patients’ tumors and their corresponding PDXs showed that xenografts resembled the primary tumors from which they derived (Figure 1a). The identification of histological subtypes enabled us to validate the models and select the chemotherapy combination to be tested in each PDX model. The choice of chemotherapy combinations was determined by clinical practice: carboplatine + paclitaxel for the five SCCs, pemetrexed + cisplatin for the eight ADCs, etoposide + cisplatin for the large-cell neuroendocrine carcinoma model, and gemcitabine + cisplatin for the sarcomatoid carcinoma. Overall, our models are not very sensitive to chemotherapy, whatever the histological subtype (Figure 1b,c) (individual data per model in Supplemental Figure S3). Only one among five SCCs responded to carboplatin + paclitaxel with tumor stabilization (Figure 1d). Interestingly, the two most chemosensitive SCC PDX models (LCIM1 and LCIM26) (Supplemental Figure S3) harbored an inactivating mutation of PTEN, in contrast to wild-type PTEN tumors (LCIM6, LCIM22 and LCIM25) (Figure 1e). Moreover, when we compared each mono-chemotherapy to the combination, we have observed that PTEN-mutated tumors are more sensitive to carboplatin (Figure 1e), a finding not reported so far in the clinic.

### 3.3. Additive Antitumor Effect of Combining Chemotherapy with an EGFR TKI in an ADC PDX with EGFR L858R Mutation

**Figure 2:**
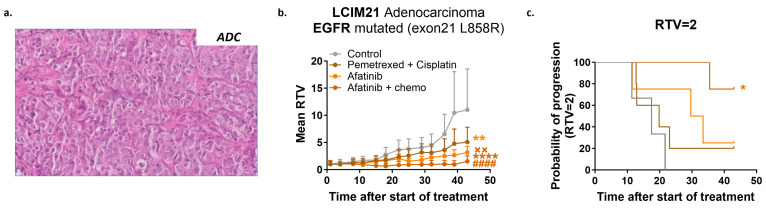
In vivo efficacy study of EGFR inhibitor ± standard chemotherapy in EGFR-mutated adenocarcinoma NSCLC PDX. (**a**) HES histological staining of PDX LCIM21 adenocarcinoma (magnification ×200). (**b**) In vivo targeting of LCIM21 PDX by afatinib (15 mg/kg, 5x/w, per os) (*n* = 4) in monotherapy (light orange line) and in combination (dark orange line) with pemetrexed (100 mg/kg, 1x/w, ip) plus cisplatin (4 mg/kg, 1x/3w, ip) (*n* = 4) (brown line); statistical analysis of the efficacy of the treatment was performed by unpaired *t*-test (Mann–Whitney test). * vs. control group, # vs. chemotherapy, x vs. afatinib. (**c**). Probability of progression after each tested treatment; the time to reach relative tumor volume RTV = 2 for each treated mouse has been calculated.

To investigate the value of combining a targeted therapy with chemotherapy in NSCLC, we first tested the antitumor effect of a clinically approved EGFR TKI, afatinib, in combination with pemetrexed + cisplatin in a PDX model of *EGFR* L858R-mutated ADC (histological analysis in Figure 2a). Looking at mean relative tumor volume (RTV), we observed in the LCIM21 model a high and significantly better efficacy of the combination of afatinib with chemotherapies (TGI = 86%) compared to afatinib (TGI = 71%) or chemotherapies (TGI = 54%) alone (Figure 2b) (unpaired *t*-test). In addition, by determining the probability of progression (two-fold increase in RTV), we observed a significant efficacy of the combination of EGFR TKI with chemotherapies compared with the control group (*p* = 0.0386), which was not found in therapies administered alone (Figure 2c) (Mantel-Cox test). In conclusion, this first experiment demonstrated that the combination of targeted therapy and chemotherapy can improve antitumor response in a PDX model of *EGFR* L858R-mutated ADC, as reported into clinical trials [6,15]. 

### 3.4. In Vivo Efficacy of Combining MEK Inhibitor with Chemotherapy in NSCLC PDX Models Showing Alterations in the MAPK Pathway

**Figure 3:**
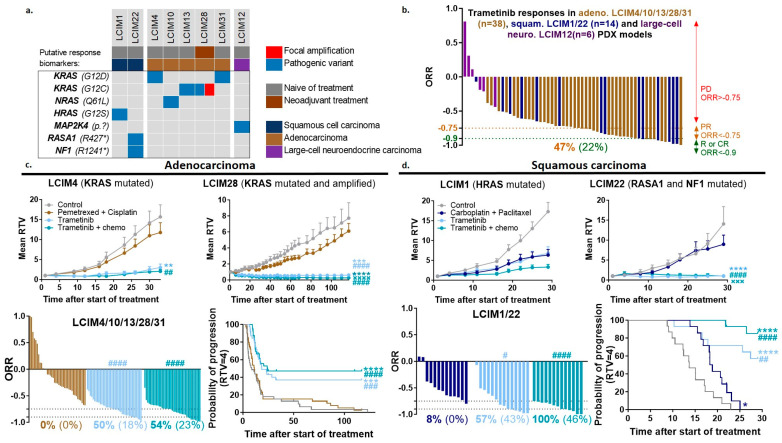
In vivo efficacy study of KRAS inhibitor ± standard chemotherapy in NSCLC PDX models showing alterations in the MAPK pathway. (**a**) Histological and genomic characteristics of PDX models with MAPK pathway alterations. (**b**) Overall response rate (ORR) to trametinib monotherapy (0.4 mg/kg, 5x/w, per os) in 8 NSCLC PDXs; a percentage in orange corresponds to an ORR lower than −0.75 and a percentage in green corresponds to an ORR lower than −0.9. In vivo targeting of adenocarcinoma NSCLC PDXs by trametinib (light blue line) in combination (turquoise blue line) with pemetrexed (100 mg/kg, 1x/w, ip) plus cisplatin (4 mg/kg, 1x/3w, ip) (brown line) (**c**) and of squamous carcinoma PDXs by trametinib (ligh blue line) in combination (turquoise blue line) with carboplatin (66 mg/kg, 1x/3w, ip) plus paclitaxel (20 mg/kg, 1x/3w, ip) (dark blue line) (**d**). Mean RTV ± SEM (*n* = 6 to 8); statistical analysis of the efficacy of the treatment was performed by unpaired *t*-test (Mann–Whitney test). * vs. control group, # vs. chemotherapy, × vs. trametinib. ORR: a percentage in bold corresponds to an ORR lower than −0.75 and a percentage in brackets correspond to an ORR lower than −0.9; statistical analysis of ORR was performed by unpaired *t*-test (Mann-Whitney test); # vs. chemotherapy. Probability of progression after each tested treatment; the time to reach relative tumor volume RTV = 4 for each treated mouse has been calculated; statistical analysis of the efficacy of the treatment was performed by Mantel–Cox test. * vs. control group, # vs. chemotherapy.

Trametinib, an MEK inhibitor, was tested alone and in combination with chemotherapy in eight NSCLC PDXs with MAPK pathway alterations. The targeting of the MAPK pathway in this study was not limited to *KRAS* mutations but extended to other genomic alterations found in our PDX models (*NRAS*, *HRAS*, *RASA1*, *MAP2K4*, *NF1*) (histological and genomic characteristics in Figure 3a). Independent of the type of mutations, treatment with trametinib alone induced a significant antitumor response, with an ORR < −0.75 in 47% of treated mice and an ORR < −0.9 in 22% of treated mice (Figure 3b). The strongest responses to trametinib were observed in LCIM22 SCC and LCIM28 ADC PDXs. Both models show a double genomic alteration in the MAPK pathway, *RASA1* and *NF1* mutations for LCIM22, and mutation and amplification of *KRAS* for LCIM28 (individual data per model in Supplemental Figure S4). When we examined ORR and probability of progression (RTV = 4), the addition of chemotherapy to trametinib appears to improve the quality of antitumor responses in SCC (57% of mice treated with trametinib have ORR < −0.75 vs. 100% of mice treated with the combination) but not in ADC (Figure 3c,d).

These results suggest that clinical trials of MAPK pathway inhibitors should not be limited to KRAS gene alterations but should be extended to all mutations resulting in the activation of the pathway. Finally, we have shown the value of using trametinib in combination with chemotherapy in SCCs with activating alterations of the MAPK pathway, which needs to be validated on a larger scale in patients.

### 3.5. In Vivo Efficacy of Combining PI3K or mTOR Inhibitors with Chemotherapy in NSCLC PDX Models Showing Alterations in the PI3K Pathway

**Figure 4:**
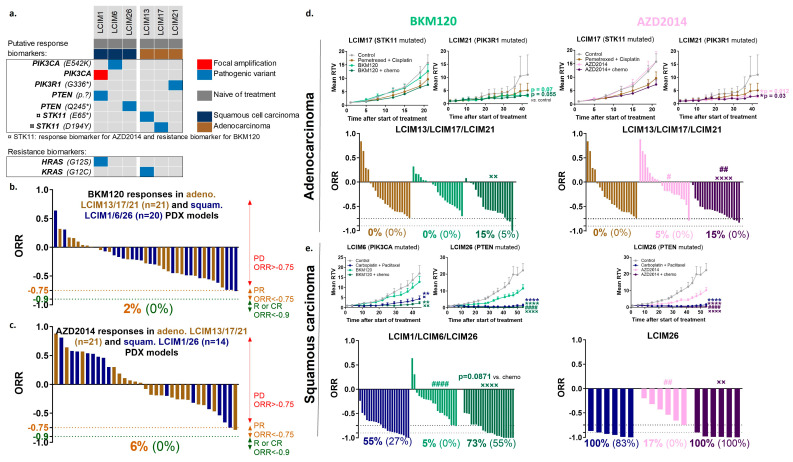
In vivo efficacy study of PI3K or mTOR inhibitors ± standard chemotherapy in NSCLC PDX models showing alterations in the PI3K pathway. (**a**) Histological and genomic characteristics of PDX models with PI3K pathway alterations. Overall response rate (ORR) to BKM120 monotherapy (15 mg/kg, 5x/w, per os) in 6 NSCLC PDXs (**b**) and to AZD2014 monotherapy (10 mg/kg, 5x/w, per os) in 5 NSCLC PDXs (**c**) (*n* = 6 to 8); a percentage in orange corresponds to an ORR lower than −0.75 and a percentage in green corresponds to an ORR lower than −0.9. In vivo targeting of adenocarcinoma NSCLC PDXs by BKM120 (green line) and AZD2014 (pink line) in combination with pemetrexed (100 mg/kg, 1x/w, ip) plus cisplatin (4 mg/kg, 1x/3w, ip) (**d**) and of squamous carcinoma PDXs in combination with carboplatin (66 mg/kg, 1x/3w, ip) plus paclitaxel (20 mg/kg, 1x/3w, ip) (dark blue line) (**e**). Mean RTV (*n* = 6 to 8); statistical analysis of the efficacy of the treatment was performed by unpaired *t*-test (Mann–Whitney test). * vs. control group. ORR: a percentage in bold corresponds to an ORR lower than −0.75 and a percentage in brackets corresponds to an ORR lower than −0.9; statistical analysis of ORR was performed by unpaired *t*-test (Mann–Whitney test); # vs. chemotherapy, × vs. BKM120 or AZD2014.

The antitumor activity of BKM120, a PI3K inhibitor, and AZD2014, an mTORC1/C2 inhibitor, was tested in our 6 NSCLC PDXs with various genetic alterations that activate the PI3K pathway (mutations of PIK3CA, PIK3R1, PTEN, STK11) (Figure 4a). Treatment with BKM120 and AZD2014 as monotherapies had no antitumor effect in our PI3K-altered PDX panel (all mutations and all histological subtypes), with ORR < −0.75 in only 2% and 6% of treated mice, respectively (Figure 4b,c). The two PDXs with a mutation in the MAPK pathway (HRAS for LCIM1 and KRAS for LCIM13), which is a marker of resistance to PI3K inhibitors, had the lowest median ORR in response to PI3K and mTOR inhibitors alone in SCC and ADC subgroups, respectively (individual data per model in Supplemental Figure S5). In ADC models, the combination of chemotherapy with BKM120 or AZD2014 does not appear to improve the antitumor efficacy of PI3K and mTOR inhibitors (ORR < −0.75 in 0% and 5% of mice treated with BKM120 or AZD2014, respectively, versus 15% and 15% of mice treated with the combination) (Figure 4d). In contrast, in our SCC models, combining chemotherapy with BKM120 or AZD2014 non-significantly improved the quality of antitumor responses (BKM120: ORR < −90 in 55% of mice treated with the combination compared to 27% of mice treated with chemotherapy alone; AZD2014: ORR < −90 in 100% of mice treated with the combination compared to 83% of mice treated with chemotherapy alone) (Figure 4e).

Similar to the results of targeting the MAPK pathway, these results demonstrate the value of combining chemotherapy with PI3K-targeted therapy in SCC. Beyond histological subtypes, it should be noted that the mutations present in our SCC models (mutations of *PTEN* and *PIK3CA* mutations) present a higher level of evidence for the use of PIK3CA pathway inhibitors than our ADC models (*PIK3R1* and *STK11* mutations, *STK11* being also a potential marker of resistance to BKM120).

Given the poor response to BKM120 and AZD2014 monotherapy in our models with PI3K pathway alterations, dual targeting of the PI3K pathway by concomitant treatment of mice with BKM120 and AZD2014 was tested (Supplemental Figure S6). Although slightly significant differences were observed between the monotherapy and dual therapy groups in the ADC and SCC models, we consider that the combination of the two inhibitors did not have a very potent additive antitumor effect (only 5% and 20% of mice treated with the combination had an ORR < −0.75 in ADCs and SCC, respectively). In contrast, an additional experiment has shown that dual targeting of the PI3K and MAPK pathways with BKM120 plus trametinib induced a better antitumor effect than dual targeting of the PI3K pathway with BKM120 plus AZD2014 in the *NF1*-mutated LCIM22 PDX model (Supplemental Figure S7).

### 3.6. In Vivo Efficacy Assessment of Targeted Therapy in NSCLC PDX Models Showing CDKN2A, MYC, or Epigenetic Alterations

**Figure 5:**
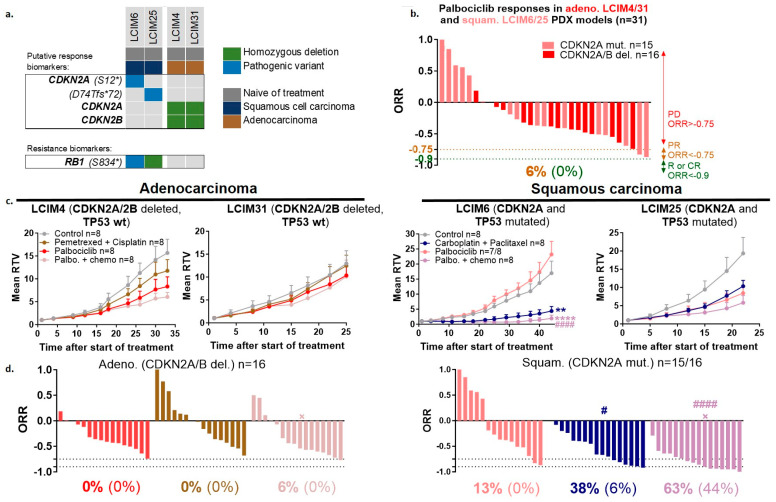
In vivo efficacy study of CDK4/6 inhibitor ± standard chemotherapy in NSCLC PDX models showing CDKN2A alterations. (**a**) Histological and genomic characteristics of PDX models with CDKN2A alterations. (**b**) Overall response rate (ORR) to palbociclib monotherapy (50 mg/kg, 5x/w, per os) in 4 NSCLC PDXs; a percentage in orange corresponds to an ORR lower than −0.75 and a percentage in green corresponds to an ORR lower than −0.9. (**c**) In vivo targeting of 4 NSCLC PDXs by palbociclib in combination with chemotherapy (for adenocarcinomas: pemetrexed (100 mg/kg, 1x/w, ip) plus cisplatin (4 mg/kg, 1x/3w, ip), for squamous carcinomas: carboplatin (66 mg/kg, 1x/3w, ip) plus paclitaxel (20 mg/kg, 1x/3w, ip)); Mean RTV ± SEM (*n* = 8); statistical analysis of the efficacy of the treatment was performed by unpaired *t*-test (Mann–Whitney test). * vs. control group, # vs. palbociclib. (**d**) ORR; a percentage in bold corresponds to an ORR lower than −0.75 and a percentage in brackets corresponds to an ORR lower than −0.9; statistical analysis of ORR was performed by unpaired *t*-test (Mann–Whitney test); # vs. palbociclib, × vs. chemotherapy.

To determine whether a *CDKN2A* mutation or homologous deletion (found in 38% of our NSCLC PDX panel) can be used as a biomarker of response to a CDK4/6 inhibitor in NSCLC, treatment with palbociclib alone or in combination with chemotherapies was carried out in two ADCs with *CDKN2A* homologous deletions and two SCCs with *CDKN2A* mutations (Figure 5a). Treatment with palbociclib alone did not induce an antitumor effect in the PDX tested, with an ORR < −0.75 only in 6% of palbociclib-treated mice (Figure 5b) (individual data per model in Supplemental Figure S8). When considering ORR, the addition of chemotherapy to palbociclib significantly improved the quality of antitumor responses in squamous-cell carcinomas (13% of palbociclib-treated mice had ORR < −0.75 versus 63% of combination-treated mice), but not in ADCs, despite the presence of RB1 alterations (a potential biomarker of palbociclib resistance) in squamous-cell carcinomas (Figure 5c,d). Finally, additional experiments have shown that treatment of PDXs displaying MYC (focal amplification) and epigenetic (mutations of ARID1A and SMARCA4) alterations using a BET bromodomain inhibitor and an EZH2 inhibitor, respectively, did not induce antitumor effects in our NSCLC PDX models (Supplemental Figure S9).

## 4. Discussion

In this article, we have evaluated whether combined chemotherapy and targeted therapy in NSCLC PDXs, both defined according to the histopathological subtypes and molecular abnormalities of the tumors, may be more efficient than chemotherapy or targeted therapy alone. In contrast to ADCs in which no significant additive effect was observed, our results show that, in squamous NSCLC, carboplatin + paclitaxel chemotherapy combined with targeted therapies directed against MAPK and PI3K pathways, or CDK4/6, were significantly more efficient than each treatment tested alone. We therefore consider that such an observation might be considered in the management of squamous NSCLC patients.

We have used for our work PDX models that are recognized to highly recapitulate the heterogeneity of human tumors and are strongly predictive of treatment responses in cancer patients [10,16]. In the NSCLC PDX panel that we developed, we observed a similar distribution of cancer subtypes, as well as similar and poor responses to standard chemotherapies in comparison to that observed in the clinics. We aim to highlight four peculiar points concerning our NSCLC PDX panel: First, in contrast to what has been reported in other cancer type PDXs [11,17,18], we did not observe that, in our series, NSCLC patients whose tumors were able to grow in mice had a shorter overall survival than patients whose tumors were not able to grow in mice (Supplemental Figure S2b); however, this may be due to the limited number of established models. Second, molecular analyses of our PDX panel were not limited to standard alterations such as *EGFR* or *KRAS* mutations, but to 571 genes (studied in the NGS panel called “DRAGON”) of interest in relation to a theranostic approach. Third, we observed that patients whose tumors expressed high levels of PDL1 significantly correspond to patients whose tumors did not grow in mice (Supplemental Figure S2c); this observation is at odds with a recent study in pancreatic cancer which reported that PDL1 expression correlated with tumor uptake in PDX models [19]. Fourth, we observed that *PTEN*-mutated tumors, exclusively observed in squamous PDXs, were more sensitive to carboplatin than wild-type *PTEN* tumors (Figure 1e). This observation contradicts previous studies showing that PTEN loss correlates with shorter recurrence-free survival in breast cancer and worse overall survival in head and neck squamous-cell carcinoma patients treated with chemotherapy [20,21]. It might therefore be of interest to evaluate the possible relationship between PTEN status in squamous NSCLC and response to carboplatin-based chemotherapy. 

The combination of chemotherapy with a targeted therapy (gefitinib or osimertinib) in NSCLC patients has been proved to be superior to chemotherapy alone in the context of *EGFR* mutations [6,15,22]. A series of molecular targeted agents have been tested in combination to standard chemotherapy in NSCLC patients, as shown in Table 1. The results of clinical non-randomized studies have been reported. Few clinical randomized studies have been reported with selumetinib (an MEK inhibitor) [23], OGX-427 (an HSP 90 inhibitor) [24], Enzastaurine (a PKC and PI3K pathways inhibitor) [25], tirapazamine (which targets hypoxic cells) [26], and different antiangiogenic compounds [27,28,29,30]. Among all these studies, mainly performed in non-squamous NSCLC, only one showed a significant difference between chemotherapy alone (cisplatin + gemcitabine) and chemotherapy + bevacizumab in favor of the combination [29]. Otherwise, no significant improvement in terms of ORR, progression-free survival, and overall survival have been observed, and in particularly after treatment with the MEK inhibitor selumetinib in metastatic pretreated *KRAS*-mutated NSCLC [23]. This point underlines the fact that the evaluation of efficient combinations in *EGFR*-wild-type NSCLC remains a real and current therapeutic challenge.

In this view, our study appears of high interest to define, according to specific molecular abnormalities of the tumors (using an NGS panel), which combinations of chemotherapy plus targeted therapy are susceptible to improving the therapeutic management of both ADC and squamous NSCLCs. In contrast to current clinical practices, such an approach also opens the possibility to evaluate the impact of the NGS-profile study of squamous NSCLC. Hence, the observation that the MEK inhibitor trametinib was also able to induce tumor regressions in *HRAS*-, *NRAS*-, and *RASA1*-mutated squamous NSCLC PDXs, in parallel to *KRAS*-mutated ADC PDXs (Figure 3), supports the enlargement of the number of NSCLC patients who could benefit from treatments including an MEK inhibitor and, consequently, appears to be of high clinical interest. The PDX model of SCC (LCIM22) that responded best to the MEK inhibitor had a double genomic alteration in the MAPK pathway, namely a *RASA1* mutation and an *NF1* mutation. These results are in accordance with a recent study showing that *RASA1* truncating mutations have a strong tendency to co-occur with NF1 truncating mutations, and that alterations in these two negative regulators of RAS signaling, when concomitant, show profound sensitivity to MEK inhibition in vitro [36]. Moreover, the PDX model of ADC (LCIM28) that highly responded to the MEK inhibitor in our study exhibited a dual alteration of the MAPK pathway with a *KRAS* mutation plus a *KRAS* amplification. Trametinib has been shown to have clinical activity in a variety of malignancies, including *KRAS*-mutated NSCLC [37,38]. However, because of the presence of compensatory signaling pathways, targeting of *MEK* alone may not achieve a complete antitumor effect [39]. Our results show that a double alteration in the MAPK pathway seems to give a therapeutic advantage to ADC and SCC patients receiving MEK inhibitors. This observation underlines, as already described [40,41] the importance of determining the presence of a *KRAS* mutation in a tumor, as well as accurately assessing KRAS expression levels that may impact potentially therapeutic KRAS-targeted therapies. 

In a similar view, it is noteworthy to note that various mutations affecting the PI3K pathway, such as *PIK3CA*, *PIK3R1*, *PTEN*, and *STK11* mutations, may define possible targeted therapies that could be combined with standard chemotherapy, even if the theranostic weight of each mutation remains to be refined. Finally, our results do not validate *CDKN2A* alterations (mutations or deletions) as predictive biomarkers of response to palbociclib alone in NSCLC. However, once again, combining the CDK4/6 inhibitor with chemotherapy improved the response to chemotherapy only in squamous PDXs. The current clinical rule of evaluating mutational/copy-number status only in ADC NSCLC needs to be re-examined in the light of our results showing that the combination of chemotherapy plus targeted therapy was significantly more efficient than monotherapies in squamous PDXs. Although we lack mechanisms to explain the efficacy of targeted therapies combined with chemotherapy in CSC, and based on our significant preclinical data, we consider that a clinical trial can be launched to assess the impact of adding NGS-targeted therapy to chemotherapy. However, adverse reactions or toxicity may also be assessed in those cancer patients. Based on what we have observed in a relevant panel of NSCLC PDXs, our data support conducting further clinical trials to hopefully improve the prognosis of NSCLC patients. 

## 5. Conclusions

In conclusion, this study has showed that, in contrast to adenocarcinomas, in squamous non-small-cell lung cancer, chemotherapy combined with targeted therapies directed at the PI3K or MAPK pathways, or against alterations in *CDNK2A* gene, was more effective than each treatment tested alone. Based on what we observed in a relevant panel of NSCLC PDXs, we suggest that the NSCLC clinical paradigm could be modified in the direction of systematically opening up NGS evaluation to squamous NSCLC, to define relevant combinations of therapies likely to improve the prognosis of patients with squamous NSCLC. Furthermore, our results suggest that the use of TKIs in combination with chemotherapy should not be limited to targeting *KRAS* or *PIK3CA* mutations, but should be extended to targeting *HRAS*, *NRAS*, *RASA1*, and *NF1* alterations for KRAS inhibitors, and *PTEN* and *PIK3R1* alterations for PI3KCA inhibitors.

## Figures and Tables

**Table 1:** Randomized and non-randomized clinical trials evaluating chemotherapy plus targeted therapies in NSCLC patients.

Chemotherapies	Molecular Targeted Agents	Rando-Mized Studies	Sub-Type of Cancer	N of Patients	Results	Ref.
**Docetaxel**	Selumetinib (MEKi)	Yes	Metastatic pretreated KRAS-mutant NSCLC	44	mPFS 5.3 months (combi) versus 2.1 months (CT alone)	[23]
**Carboplatin + pemetrexed**	OGX-427 (HSP 27 mRNA)	Yes	Untreated metastatic non-squamous NSCLC	155	ORR, mPFS, and mOS: NS	[24]
**Cisplatin + pemetrexed**	Enzastaurin (PKC & AKT pathways)	Yes	Chemonaive metastatic NSCLC	22	Early interruption due to combination toxicity	[25]
**Cisplatin + gemcitabine**	Sorafenib (antiangiogenic agent)	Yes	Metastatic NSCLC	30	ORR, mPFS, and mOS: NS	[27]
**Cisplatin + gemcitabine**	Sorafenib (antiangiogenic agent)	Yes	Untreated metastatic non-squamous NSCLC	772	PFS: NS	[28]
**Cisplatin + gemcitabine**	Bevacizumab (antiangiogenic agent)	Yes	Untreated metastatic non-squamous NSCLC	1043	Combi > CT alone (*p* = 0.03)	[29]
**Pemetrexed**	Sunitinib (antiangiogenic agent)	Yes	Second-line metastatic NSCLC	130	High toxicity of combination impacted PFS and OS	[30]
**Carboplatin + paclitaxel**	Tirapazamine (hypoxic cells)	Yes	Metastatic NSCLC	367	Early interruption due to combination toxicity	[26]
**Docetaxel**	Celecoxib (COX-2i)	No	Recurrent NSCLC	56	ORR 11%	[31]
**Pemetrexed + docetaxel**	Apatinib (antiangiogenic TKI)	No	Refractory metastatic NSCLC	20	ORR 30% (PR)	[32]
**Docetaxel**	Apatinib (antiangiogenic TKI)	No	Metastatic non-squamous NSCLC	14	ORR 33%	[33]
**Pemetrexed**	Luminespib (HSP90i)	No	Metastatic non-squamous NSCLC	13	ORR 14%	[34]
**Carboplatin + pemetrexed**	Metformine	No	Treatment-naive metastatic non-squamous NSCLC	/	mPFS 4.5 months	[35]

## Data Availability

All data are already shared in the paper.

## Supplementary Materials

The following supporting information can be downloaded at: https://www.mdpi.com/article/10.3390/cancers16162785/s1. Table S1: Targeted NGS panel gene list. Table S2: Clinical and molecular characteristics of all NSCLC patients (n = 31; univariate analysis) and in vivo tumor take rate (%). Table S3: Molecular characteristics of NSCLC PDX panel. Figure S1: Classification of anti-tumor responses. Figure S2: Development of a NSCLC PDX panel. Figure S3: Overall response rate (ORR) per model to standard chemotherapies. Figure S4: Overall response rate (ORR) per model to KRAS inhibitor ± standard chemotherapy in NSCLC PDX models showing alterations in the MAPK pathway. Figure S5: Overall response rate (ORR) per model to PI3K or mTOR inhibitors ± standard chemotherapy in NSCLC PDX models showing alterations in the PI3K pathway. Figure S6: In vivo efficacy of dual targeting of the PI3K pathway in NSCLC PDX models. Figure S7: In vivo efficacy of dual targeting of the PI3K and MAPK pathways or dual targeting of the PI3K pathway in NF1-mutated NSCLC PDX model. Figure S8: Overall response rate (ORR) per model to CDK4/6 inhibitor ± standard chemotherapy in NSCLC PDX models showing CDKN2A alterations. Figure S9: In vivo efficacy study of MYC targeting and epigenetics targeting in NSCLC PDX models with genomic alterations.
