# Supplementary material for: Evaluation of Combined Chemotherapy and Genomic-Driven Targeted Therapy in Patient-Derived Xenografts Identifies New Therapeutic Approaches in Squamous Non-Small-Cell Lung Cancer Patients
Source: Cancers (Basel). 2024 Aug 7;16(16):2785. doi: 10.3390/cancers16162785 (PMC11352497; doi:10.3390/cancers16162785)

Supplementary

Evaluation of combined chemotherapy and genomic-driven targeted therapy in Patient-Derived Xenografts identifies new therapeutic approaches in squamous non-small-cell-lung cancer patients

Table of contents

Supplementary Table S1: Targeted NGS panel gene list..2

Supplementary Table S2: Clinical and molecular characteristics of all NSCLC patients (n=31; univariate analysis) and *in vivo* tumor take rate (%). 3

Supplementary Table S3: Molecular characteristics of NSCLC PDX panel. 4

....4

Supplementary Figure S1: Classification of anti-tumor responses 5

Supplementary Figure S2: Development of a NSCLC PDX panel...6

Supplementary Figure S3: Overall response rate (ORR) per model to standard chemotherapies.................................7

Supplementary Figure S4: Overall response rate (ORR) per model to KRAS inhibitor +/- standard chemotherapy in NSCLC PDX models showing alterations in the MAPK pathway. 8

Supplementary Figure S5: Overall response rate (ORR) per model to PI3K or mTOR inhibitors +/- standard chemotherapy in NSCLC PDX models showing alterations in the PI3K pathway..9

Supplementary Figure S6: In vivo efficacy of dual targeting of the PI3K pathway in NSCLC PDX models.....................10

Supplementary Figure S7: *In vivo* efficacy of dual targeting of the PI3K and MAPK pathways or dual targeting of the PI3K pathway in NF1-mutated NSCLC PDX model. 11

Supplementary Figure S8: Overall response rate (ORR) per model to CDK4/6 inhibitor +/- standard chemotherapy in NSCLC PDX models showing CDKN2A alterations. 12

Supplementary Figure S9: *In vivo* efficacy study of MYC targeting and epigenetics targeting in NSCLC PDX models with genomic alterations. 13

Supplementary Table S1: Targeted NGS panel gene list.

ABCB1	BCL10	CDC25C	DDX41	FAT1	HNF1A	MAP3K1	NFKBIE	PPP2R1A	RXRA	STAG2	WASF2
ABCG2	BCL11A	CDC27	DHX15	FAT2	HOXB13	MAPK1	NIPBL	PPP6C	SAMD9	STAT3	WASF3
ABHD5	BCL11B	CDH1	DICER1	FAT4	HRAS	MAX	NOTCH1	PRDM1	SAMD9L	STAT5B	WDR74
ABI1	BCL2	CDK12	DNAJC21	FBXO11	ID3	MBD4	NOTCH2	PREX2	SARAF	STAT6	WRN
ABI2	BCL2L1	CDK4	DNMT1	FBXW7	IDH1	MCL1	NOTCH3	PRIM2	SBDS	STK11	WT1
ABI3	BCL2L12	CDK6	DNMT3A	FGF10	IDH2	MDM2	NOTCH4	PRKCI	SDHA	STMN2	XPO1
ABL1	BCL6	CDK8	DPF1	FGF19	IGF1R	MDM4	NPM1	PRKD1	SDHB	SUFU	XRCC1
ACTL6A	BCL7A	CDKN1A	DPF2	FGF3	IGF2R	MECOM	NQO1	PRKD2	SDHC	SUZ12	XRCC2
ACTL6B	BCL7B	CDKN1B	DPF3	FGF4	IKBKAP	MED1	NR2F2	PRPF8	SDHD	SYF2	XRCC3
ACVR1	BCL7C	CDKN2A	DPYD	FGF6	IKZF1	MED12	NRAS	PRSS8	SETBP1	TAF1	ZDHHC19
ACVR1B	BCLAF1	CDKN2B	DROSHA	FGFR1	IKZF3	MED16	NSD1	PTCH1	SETD2	TAF1L	ZFHX3
ACVR2A	BCOR	CDKN2C	EBF1	FGFR2	IL6ST	MEF2B	NSD2	PTEN	SETDB1	TBC1D12	ZFP36L1
ACVR2B	BCORL1	CEBPA	EGFR	FGFR3	ING1	MEF2C	NT5C2	PTPN11	SF3B1	TBL1XR1	ZFP36L2
ADGRB3	BIRC2	CHD1	EIF1AX	FGFR4	INPP4B	MEN1	NTHL1	PTPRB	SF3B2	TBR1	ZIC1
ADGRG6	BIRC3	CHD2	ELANE	FH	IRF2	MET	NUMA1	PTPRD	SGK1	TBX3	ZMYM3
AGTR2	BLM	CHD3	ELF3	FLT1	IRF4	MFHAS1	NUP214	PTPRT	SH2B3	TCF12	ZMYM4
AJUBA	BMI1	CHD4	EMSY	FLT3	IRF8	MGA	NUP93	PXDNL	SLC1A2	TCF3	ZNF143
AKAP9	BMPR2	CHD6	EP300	FLT4	IRS2	miR-142	OTX2	QKI	SLC29A1	TCF4	ZNF292
AKT1	BRAF	CHD8	EP400	FOXA1	ITPKB	MITF	PALB2	RAB40A	SLC2A9	TCF7L2	ZNF471
AKT2	BRCA1	CHD9	EPHA2	FOXL2	JAK1	MLH1	PAX5	RAC1	SLIT2	TDG	ZNF750
AKT3	BRCA2	CHEK1	EPHA3	FOXO1	JAK2	MLH3	PAX6	RAD21	SLX4	TENT5C	ZRSR2
ALDOA	BRCC3	CHEK2	EPHA5	FOXP1	JAK3	MLLT4	PBRM1	RAD50	SMAD2	TERC	
ALK	BRD7	CIC	EPHA7	FOXR2	KANSL1	MN1	PCBP1	RAD51	SMAD3	TERT	
ALPK2	BRD9	CIITA	EPHB1	FRG1BP	KBTBD4	MPL	PDGFRA	RAD51B	SMAD4	TET1	
AMER1	BRIP1	CITED2	EPHB2	FRS2	KDM5C	MRE11	PDGFRB	RAD51C	SMARCA2	TET2	
ANKRD26	BRK1	CNOT9	EPPK1	FSHR	KDM6A	MSH2	PGR	RAD51D	SMARCA4	TET3	
APAF1	BTG1	COQ6	ERBB2	FUBP1	KDR	MSH3	PHF10	RAD54L	SMARCB1	TGFBR2	
APC	BTK	CRBN	ERBB3	GAS8-AS1	KEAP1	MSH6	PHF6	RAF1	SMARCC1	THAP12	
APC2	C19MC	CREBBP	ERBB4	GATA1	KIT	MST1R	PHGDH	RASA1	SMARCC2	THBS1	
AR	CALR	CRKL	ERBIN	GATA2	KLF2	MTHFR	PHOX2B	RASA2	SMARCD1	THSD7B	
ARAP3	CAND1.11	CSF1R	ERCC2	GATA3	KLF4	MTOR	PIK3C2B	RB1	SMARCD2	TLR4	
ARFRP1	CARD11	CSF3R	ESR1	GATA6	KLHL6	MUTYH	PIK3CA	RBBP6	SMARCD3	TNF	
ARHGAP35	CASP8	CSNK1A1	ETNK1	GFI1	KMT2A	MYB	PIK3CB	RBM10	SMARCE1	TNFAIP3	
ARID1A	CBFB	CTCF	ETV6	GLI1	KMT2B	MYC	PIK3CG	RECQL4	SMC1A	TNFRSF14	
ARID1B	CBL	CTNNA1	EXT1	GLI2	KMT2C	MYCL	PIK3R1	RET	SMC3	TP53	
ARID2	CBLB	CTNNB1	EXT2	GNA11	KMT2D	MYCN	PIK3R2	RHEB	SMO	TP53BP1	
ARID5B	CBLC	CTNND2	EZH2	GNA13	KRAS	MYD88	PIM1	RHOA	SMUG1	TP73	
ASXL1	CCDC107	CTPS1	FADD	GNAQ	LCK	MYO3A	PKD1	RICTOR	SNCAIP	TRAF2	
ASXL2	CCND1	CUL4B	FAM213A	GNAS	LDLRAP1	MYOD1	PLCB4	RIMS1	SNX25	TRAF3	
ATM	CCND2	CUX1	FANCA	GRIN2A	LEPROTL1	NAF1	PLCG1	RIT1	SOCS1	TRRAP	
ATR	CCND3	CXCR4	FANCB	GSTP1	LINC02008	NAV3	PLCG2	RMRP	SOX10	TSC1	
ATRX	CCNE1	CYFIP1	FANCC	H3F3A	LOC101927630	NBN	PLEKHS1	RNF169	SOX2	TSC2	
AURKA	CD274	CYFIP2	FANCD2	H3F3B	LYN	NCKAP1	PMS1	RNF43	SOX9	U2AF1	
AXIN1	CD28	CYP1A1	FANCE	HIST1H1B	LZTR1	NCKAP1L	PMS2	ROBO1	SPEN	U2AF2	
AXIN2	CD36	CYP2D6	FANCF	HIST1H3B	MALAT1	NCOR1	POLD1	ROBO2	SPOP	UBE2K	
AXL	CD58	DAXX	FANCG	HIST1H3C	MALT1	NCOR2	POLE	ROS1	SRCAP	UBR5	
B2M	CD70	DCK	FANCI	HIST2H3C	MAP2K1	NEAT1	POLE2	RPL22	SRP72	VEGFA	
BAP1	CD79A	DCTD	FANCL	HLA-A	MAP2K2	NF1	POLR2D	RPL5	SRSF2	VHL	
BARD1	CD79B	DDR2	FANCM	HLA-B	MAP2K4	NF2	POT1	RPTOR	SS18	WAS	
BC040327	CDA	DDX3X	FAS	HLA-C	MAP2K7	NFE2L2	PPM1D	RUNX1	STAG1	WASF1	

Supplementary Table S2: Clinical and molecular characteristics of all NSCLC patients (n=31; univariate analysis) and *in vivo* tumor take rate (%).

Patients and tumor characteristics	Patients (n)	Tumor take rate (%)	P
Age			
< 60 years	9	67	NS
> 60 years	22	55	
Gender			
Male	18	61.1	NS
Female	13	38.5	
Tobacco°			
Yes	27	51.9	NS
No	4	50.0	
TNM			
T1	5	40.0	
T2	10	70.0	
T3	12	30.8	
T4	4	75.0	NS
N0	18	61.1	
N1	7	42.9	
N2	5	40.0	
Nx	1	/	
Histology			
Adenocarcinoma	16	50.0	
Squamous cell carcinoma	10	50.0	NS
Sarcomatoid carcinoma	1	100.0	
Large-cell neuroendocrine carcinoma	4	50.0	

Supplementary Table S3: Molecular characteristics of NSCLC PDX panel.

PDX identity	Histology	Mutations					Focal amplifications	Homozygous deletions	TMB	MSI
		Gene	Chr	cDNA change	AA change (one letter code)	Frequence%				
LCIM1	Squamous cell carcinoma	HRAS	11	c.34G>A	p.(G12S)	98	PIK3CA	TP53 (loss), PTEN (loss)	High	Low
		TP53	17	c.872del	p.(K291Rfs*54)	92				
		RB1	13	c.1757del	p.(L586fs)	91				
		PTEN	10	c.635-1G>A	p.?	87				
LCIM4	Adenocarcinoma	ARID1A	1	c.5047G>T	p.(E1683*)	74		CDKN2A, CDKN2B	ND	Low
		KRAS	12	c.35G>A	p.(G12D)	66				
LCIM5	Sarcomatoid carcinoma	FAT2	5	c.4900G>C	p.(D1634H)	100	YAP, BIRC2, BIRC3	ATM	High	Low
		SMAD4	18	c.1619T>G	p.(L540R)	100				
		TP53	17	c.375+1del	p.?	95				
		DDX3X	X	c.1873G>T	p.(G625*)	73				
		POT1	7	c.1405A>T	p.(K469*)	63				
		FANCM	14	c.4672+3A>T	p.?	61				
		KDR	4	c.3196C>T	p.(R1066C)	38				
LCIM6	Squamous cell carcinoma	RB1	13	c.2501C>G	p.(S834*)	100	TERT		High	Low
		NOTCH1	9	c.1477G>T	p.(E493*)	99				
		CDKN2A	9	c.35C>A	p.(S12*)	99				
		TP53	17	c.615T>A	p.(Y205*)	97				
		FBXW7	4	c.1503_1525del	p.(A502*)	44				
		PIK3CA	3	c.1624G>A	p.(E542K)	14				
LCIM10	Adenocarcinoma	APC	chr5	c.531+3A>T	p.?	100	CCNE1 (focal gain)		Low	Low
		ATRX	chrX	c.6388A>T	p.(N2130Y)	100				
		NRAS	chr1	c.182A>T	p.(Q61L)	100				
		SMC1A	chrX	c.3229G>T	p.(V1077L)	100				
		TP53	chr17	c.363_375+2del	p.?	88				
		NOTCH4	chr6	c.3056G>T	p.(G1019V)	66				
		NAV3	chr12	c.2572G>T	p.(D858Y)	61				
		CHD6	chr20	c.4978G>T	p.(E1660*)	50				
		MYO3A	chr10	c.121A>T	p.(K41*)	48				
LCIM12	Large-cell neuroendocrine carcinoma	TP53	chr17	c.796G>A	p.(G266R)	100	FGFR1 (focal gain), MYC (focal gain)	TP53, MAP2K4	Low	Low
		KEAP1	chr19	c.502G>T	p.(V168F)	100				
		SMARCA4	chr19	c.3928G>T	p.(E1310*)	100				
		PTCH1	chr9	c.2866A>G	p.(M956V)	100				
		APC	chr5	c.2710A>T	p.(R904*)	100				
		ZFHX3	chr16	c.4507C>T	p.(Q1503*)	66				
		FLT3	chr13	c.2478G>T	p.(K826N)	51				
		MAP2K4	chr17	c.628_666+4del	p.?	13				
LCIM13	Adenocarcinoma	KEAP1	chr19	c.601C>T	p.(Q201*)	100	MYC		Low	Low
		KRAS	chr12	c.34G>T	p.(G12C)	100				
		STK11	chr19	c.193G>T	p.(E65*)	100				
		NAV3	chr12	c.1789T>A	p.(C597S)	100				
		APC	chr5	c.5053del	p.(G1685Kfs*24)	95				
		HIST1H1B	chr6	c.593C>G	p.(P198R)	72				
		MYCN	chr2	c.1023T>A	p.(S341R)	66				
		TP53	chr17	c.731G>C	p.(G244A)	59				
		CDK12	chr17	c.3592G>T	p.(E1198*)	35				
LCIM17	Adenocarcinoma	TP53	chr17	c.772G>T	p.(E258*)	100	MYC, CRKL		Low	Low
		STK11	chr19	c.580G>T	p.(D194Y)	100				
		JAK2	chr9	c.1968G>T	p.(Q656H)	100				
		IRS2	chr13	c.1997G>T	p.(G666V)	100				
		FAT1	chr4	c.9854G>T	p.(G3285V)	100				
		EPHA3	chr3	c.754G>T	p.(V252L)	99				
LCIM18	Large-cell neuroendocrine carcinoma	EIF1AX	chrX	c.17-2A>G	p.?	100	MYCL, PIM1		ND	Low
LCIM20	Adenocarcinoma	WHSC1	chr4	c.2725C>T	p.(Q909*)	100			High	Low
		TP53	chr17	c.528C>G	p.(C176W)	100				
		TP53BP1	chr15	c.439G>T	p.(E147*)	50				
		TAF1L	chr9	c.742_743del	p.(E248Tfs*39)	43				
		SETDB1	chr1	c.3738G>A	p.(W1246*)	24				
LCIM21	Adenocarcinoma	TP53	chr17	c.814G>T	p.(V272L)	99	MYC (focal gain)		Low	Low
		RB1	chr13	c.769C>T	p.(Q257*)	99				
		CHD3	chr17	c.4072+1G>T	p.?	99				
		PIK3R1	chr5	c.1006G>T	p.(G336*)	62				
		EGFR	chr7	c.2573T>G	p.(L858R)	31				
LCIM22	Squamous cell carcinoma	FAT1	chr4	c.8689C>T	p.(Q2897*)	100			High	Low
		CDKN2A	chr9	c.238C>T	p.(R80*)	100				
		RASA1	chr5	c.1279C>T	p.(R427*)	98				
		PREX2	chr8	c.1125G>A	p.(W375*)	68				
		DNMT3A	chr2	c.430G>T	p.(G144*)	64				
		NF1	chr17	c.3721C>T	p.(R1241*)	57				
		TP53	chr17	c.254del	p.(P85Lfs*38)	36				
		TP53	chr17	c.310C>T	p.(Q104*)	35				
		TERT	chr5	c.-124C>T	p.?	26				
LCIM25	Squamous cell carcinoma	TP53	chr17	c.458C>A	p.(P153H)	100	MYC (focal gain)	RB1 (loss)	Low	Low
		TP53	chr17	c.422G>T	p.(C141F)	100				
		KMT2D	chr12	c.4885del	p.(S1629Lfs*93)	97				
		CDKN2A	chr9	c.219del	p.(D74Tfs*72)	94				
		ASXL2	chr2	c.649C>T	p.(Q217*)	50				
LCIM26	Squamous cell carcinoma	PTEN	chr10	c.733C>T	p.(Q245*)	99		RB1	Low	Low
		TP53	chr17	c.1169del	p.(P390Lfs*32)	97				
LCIM28	Adenocarcinoma	ARID1A	chr1	c.1753C>T	p.(Q585*)	100	KRAS	CDKN2A, CDKN2B	High	Low
		TP53	chr17	c.313G>T	p.(G105C)	100				
		KRAS	chr12	c.34G>T	p.(G12C)	66				
		ROBO1	chr3	c.1343-1G>T	p.?	55				
LCIM31	Adenocarcinoma	SMAD4	chr18	c.1609_1612del	p.(D537Kfs*14)	92		CDKN2A, CDKN2B	High	Low
		KRAS	chr12	c.35G>A	p.(G12D)	62				

Supplementary Figure S1: Classification of anti-tumor responses into progressive disease, partial response, stable disease and complete response based on calculation of % of change and TGI.

An increase in tumor volume of at least a 35% with a TGI < 50% identified progressive disease, an increase in tumor volume of at least a 35% with a TGI > 50% identified partial response, volumes changes between +35% and −50% were considered as stable disease and a decrease in tumor volume of at least 50% was classified as complete response.

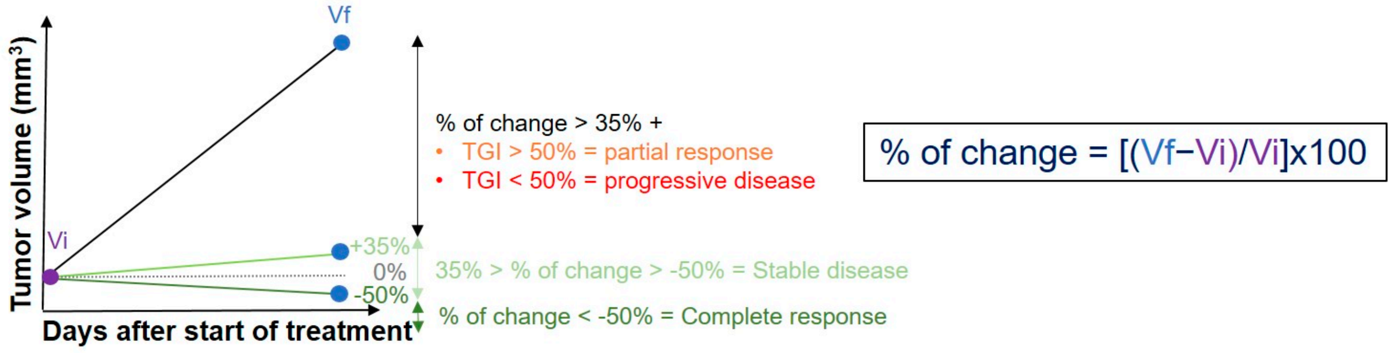

Supplementary Figure S2: Development of a NSCLC PDX panel. a. Representativeness of histological subtypes in the developed NSCLC PDX panel. Prognostic value of the *in vivo* tumor take rate on the probability of survival (b.) and on the PDL1 expression in tumors cells (c.) of corresponding NSCLC patients; unpaired t-test (Mann-Whitney test).

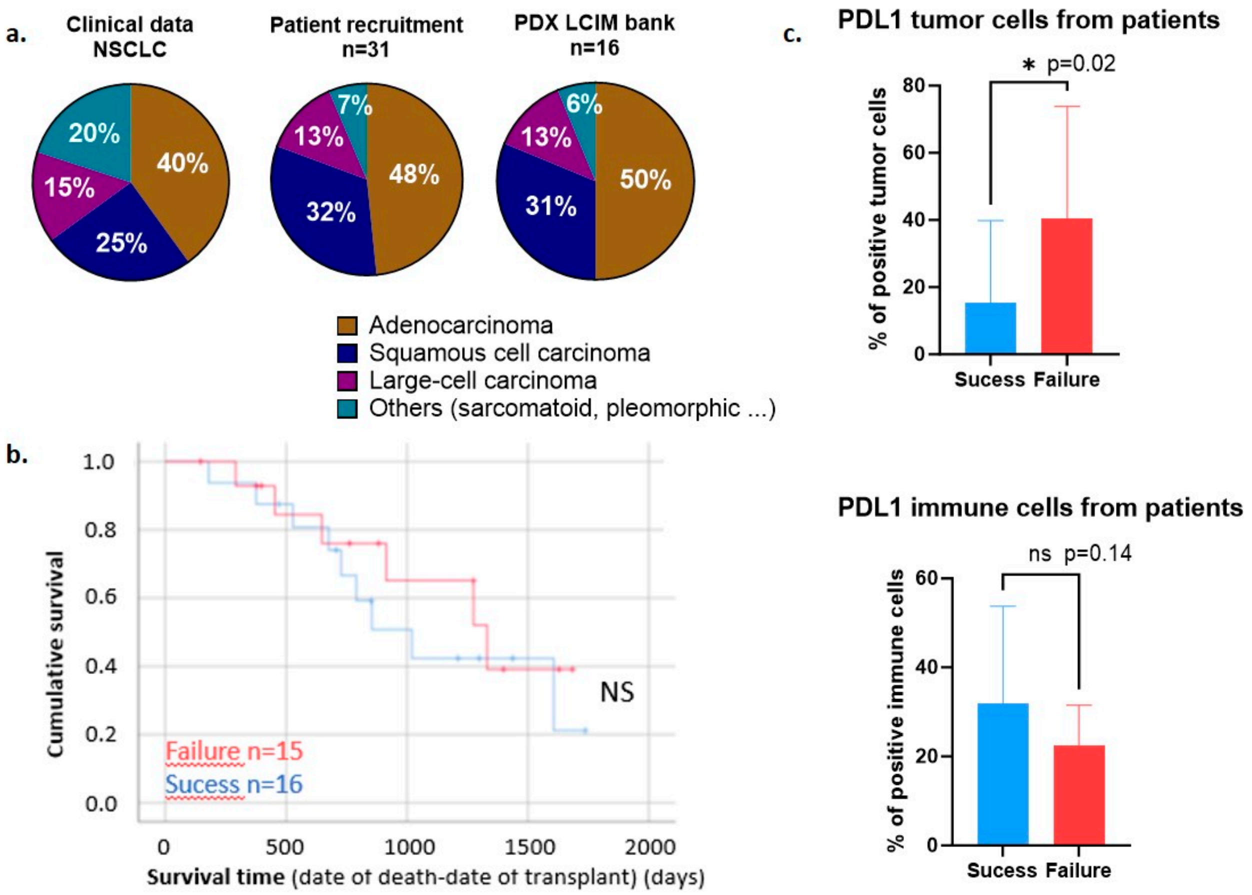

Supplementary Figure S3: Overall response rate (ORR) per model to standard chemotherapies. a. Median ORR to pemetrexed (100 mg/kg, 1x/w, ip) plus cisplatin (4 mg/kg, 1x/3w, ip) in 8 adenocarcinoma PDXs. b. Median ORR to carboplatin (66mg/kg, 1x/3w, ip) plus paclitaxel (20 mg/kg, 1x/3w, ip) in 5 squamous carcinoma PDXs. The percentage in bold correspond to an ORR lower than −0.75 and the percentage in brackets correspond to an ORR lower than −0.9; statistical analysis of ORR was performed by unpaired t-test (Mann-Whitney test); # vs. chemotherapy.

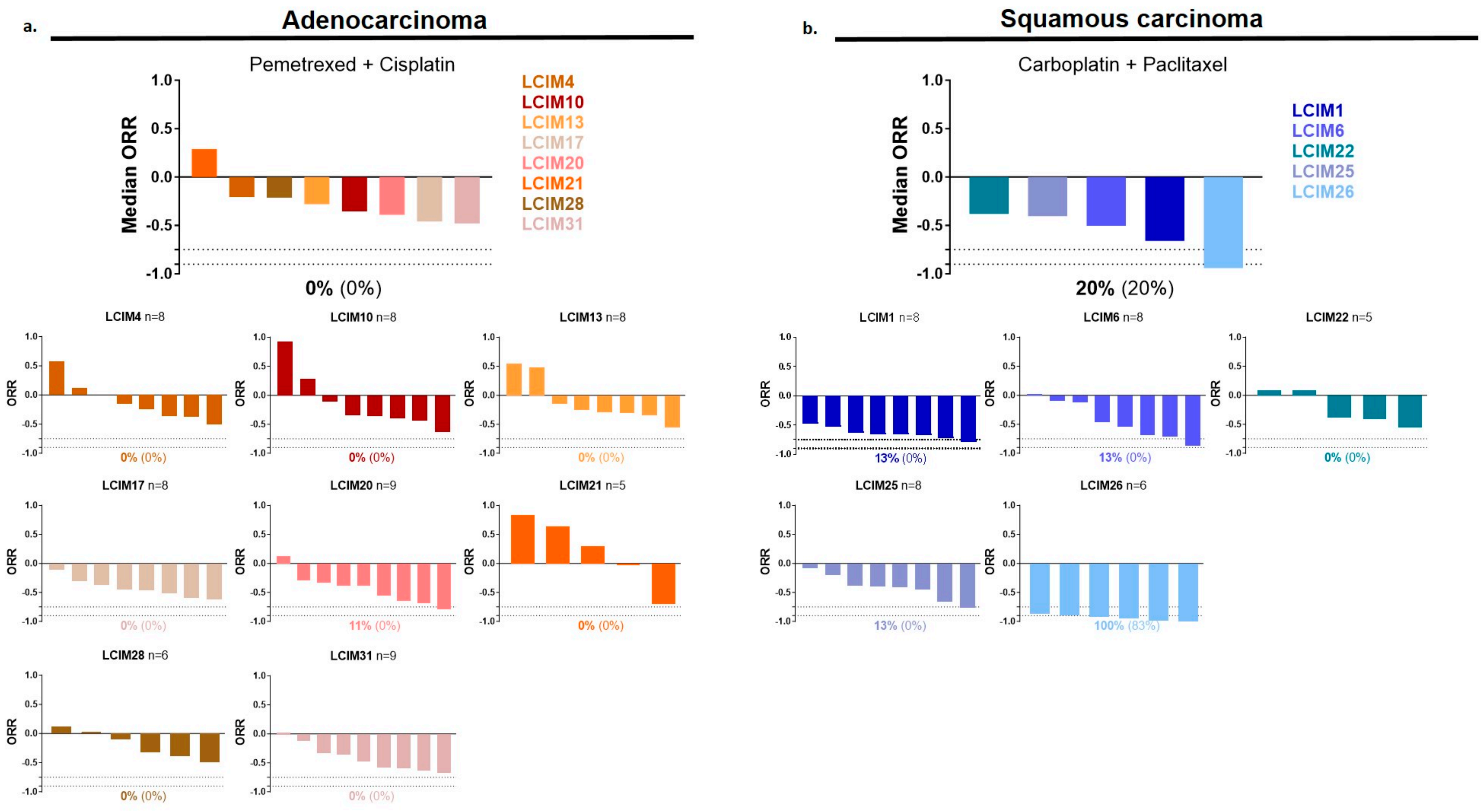

Supplementary Figure S4: Overall response rate (ORR) per model to KRAS inhibitor +/- standard chemotherapy in NSCLC PDX models showing alterations in the MAPK pathway. a. Median ORR to trametinib (0.4 mg/kg, 5x/w, *per os*) in combination with pemetrexed (100 mg/kg, 1x/w, ip) plus cisplatin (4 mg/kg, 1x/3w, ip) in 5 adenocarcinoma PDXs. b. Median ORR to trametinib (0.4 mg/kg, 5x/w, *per os*) in combination with carboplatin (66mg/kg, 1x/3w, ip) plus paclitaxel (20 mg/kg, 1x/3w, ip) in 2 squamous carcinoma PDXs. The percentage in bold correspond to an ORR lower than −0.75 and the percentage in brackets correspond to an ORR lower than −0.9; statistical analysis of ORR was performed by unpaired t-test (Mann-Whitney test); # vs. chemotherapy.

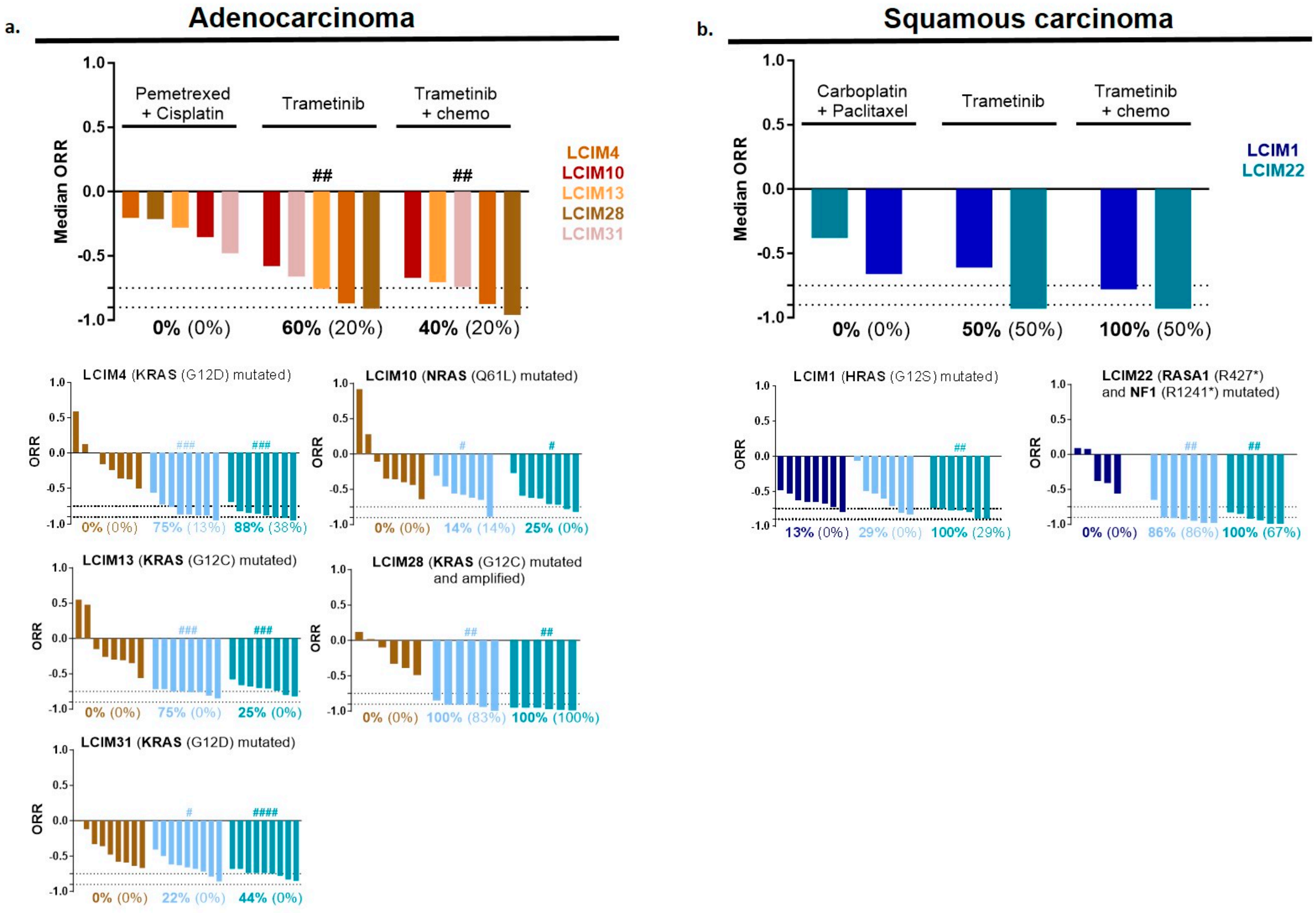

Supplementary Figure S5: Overall response rate (ORR) per model to PI3K or mTOR inhibitors +/- standard chemotherapy in NSCLC PDX models showing alterations in the PI3K pathway. a. Median ORR to BKM120 (15 mg/kg, 5x/w, *per os*) and to AZD2014 (10 mg/kg, 5x/w, *per os*) in combination with pemetrexed (100 mg/kg, 1x/w, ip) plus cisplatin (4 mg/kg, 1x/3w, ip) in 3 adenocarcinoma PDX models. b. Median ORR to BKM120 (15 mg/kg, 5x/w, *per os*) and to AZD2014 (10 mg/kg, 5x/w, *per os*) in combination with carboplatin (66mg/kg, 1x/3w, ip) plus paclitaxel (20 mg/kg, 1x/3w, ip) in 3 squamous carcinoma PDX models. The percentage in bold correspond to an ORR lower than −0.75 and the percentage in brackets correspond to an ORR lower than −0.9; statistical analysis of ORR was performed by unpaired t-test (Mann-Whitney test); # vs. chemotherapy, × vs. BKM120 or AZD2014.

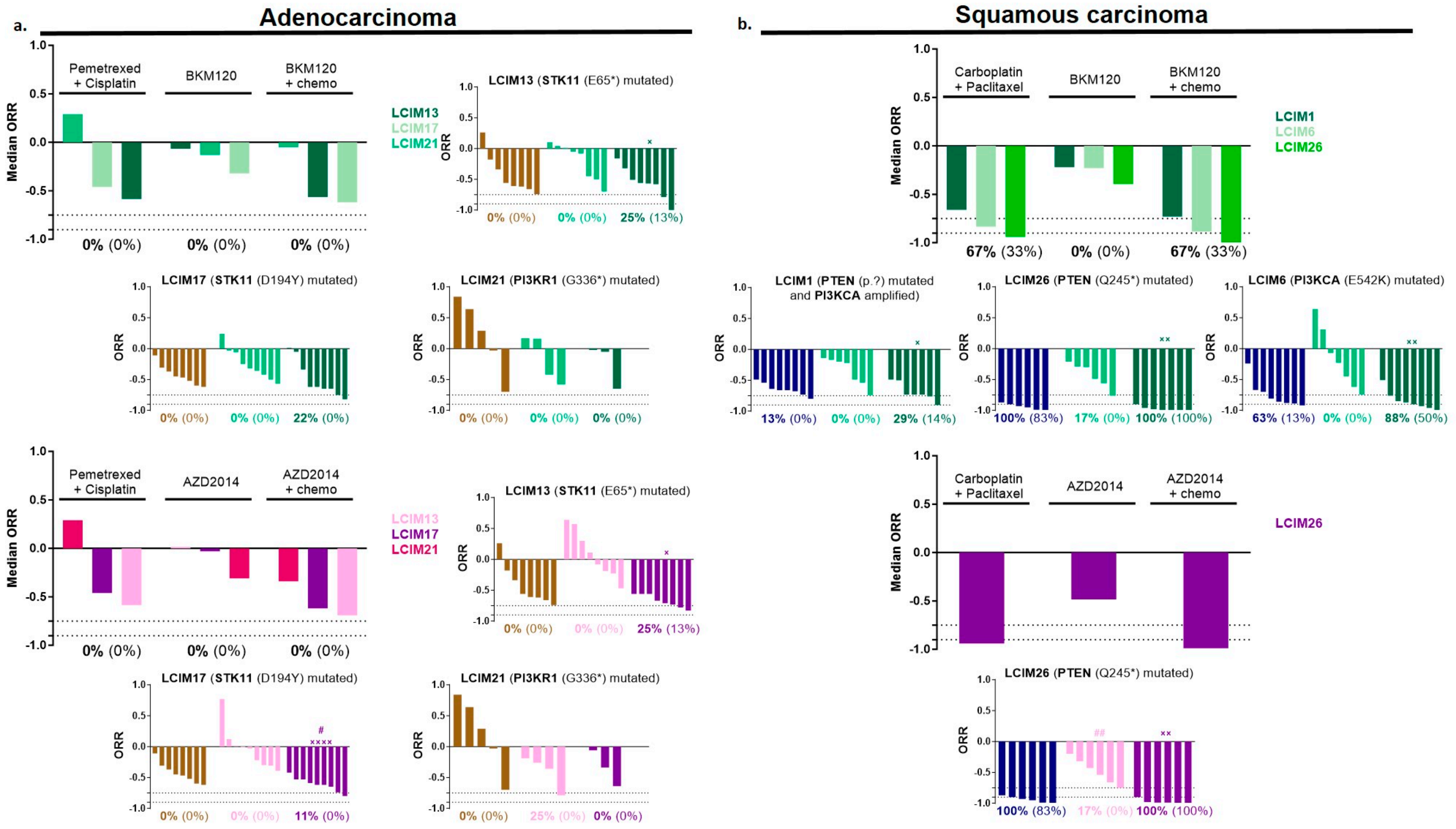

Supplementary Figure S6: In vivo efficacy of dual targeting of the PI3K pathway in NSCLC PDX models. *In vivo* targeting of 3 adenocarcinoma (a.) and 2 squamous carcinoma (b.) NSCLC PDXs by BKM120 (15 mg/kg, 5x/w, *per os*) plus AZD2014 (10 mg/kg, 5x/w, *per os*). Mean RTV +/- SEM; statistical analysis of the efficacy of the treatment was performed by unpaired t-test (Mann-Whitney test). * vs. control group, # vs. chemotherapy group. Overall response rate (ORR); the percentage in bold correspond to an ORR lower than −0.75 and the percentage in brackets correspond to an ORR lower than −0.9; statistical analysis of ORR was performed by unpaired t-test (Mann-Whitney test); # vs. BKM120, × vs. AZD2014.

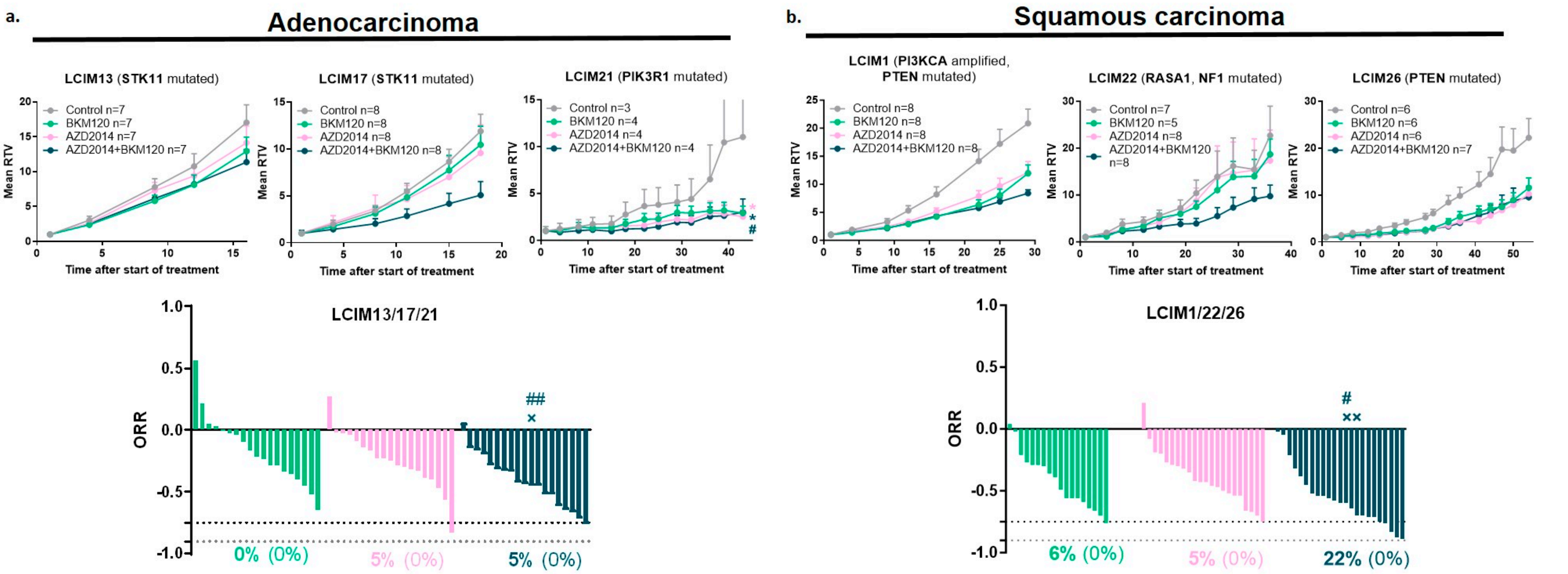

Supplementary Figure S7: *In vivo* efficacy of dual targeting of the PI3K and MAPK pathways or dual targeting of the PI3K pathway in NF1-mutated NSCLC PDX model. *In vivo* targeting of a NF1-mutated squamous carcinoma NSCLC PDX by BKM120 (15 mg/kg, 5x/w, *per os)* plus trametinib (0.4 mg/kg, 5x/w, *per os*) (a.) or BKM120 (15 mg/kg, 5x/w, *per os*) plus AZD2014 (10 mg/kg, 5x/w, *per os*) (b.). Mean RTV +/- SEM; Overall response rate (ORR); the percentage in bold correspond to an ORR lower than −0.75 and the percentage in brackets correspond to an ORR lower than −0.9; statistical analysis of ORR was performed by unpaired t-test (Mann-Whitney test); # vs. BKM120, × vs. trametinib.

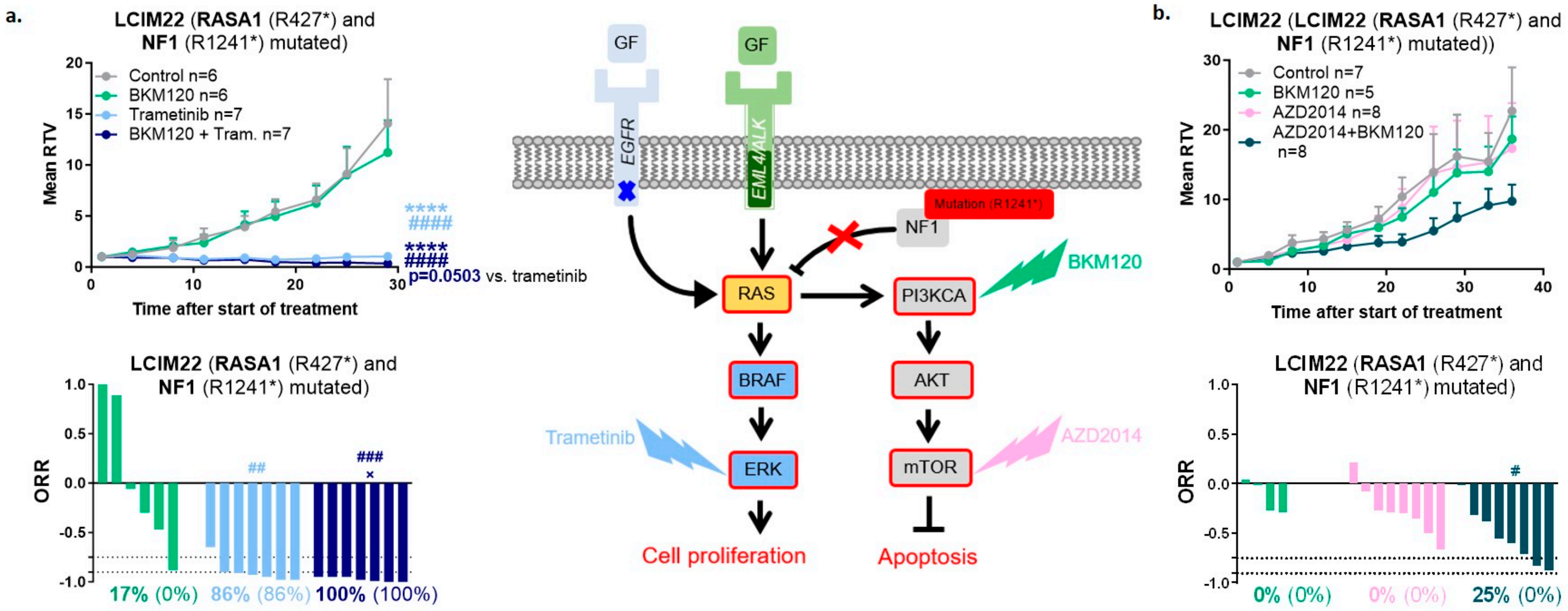

Supplementary Figure S8: Overall response rate (ORR) per model to CDK4/6 inhibitor +/- standard chemotherapy in NSCLC PDX models showing CDKN2A alterations. a. Median ORR to palbociclib (50 mg/kg, 5x/w, *per os*) in combination with pemetrexed (100 mg/kg, 1x/w, ip) plus cisplatin (4 mg/kg, 1x/3w, ip) in 2 adenocarcinoma PDXs. b. Median ORR to palbociclib (50 mg/kg, 5x/w, *per os*) in combination with carboplatin (66mg/kg, 1x/3w, ip) plus paclitaxel (20 mg/kg, 1x/3w, ip) in 2 squamous carcinoma PDXs. The percentage in bold correspond to an ORR lower than −0.75 and the percentage in brackets correspond to an ORR lower than −0.9; statistical analysis of ORR was performed by unpaired t-test (Mann-Whitney test); # vs. palbociclib, × vs. chemotherapy.

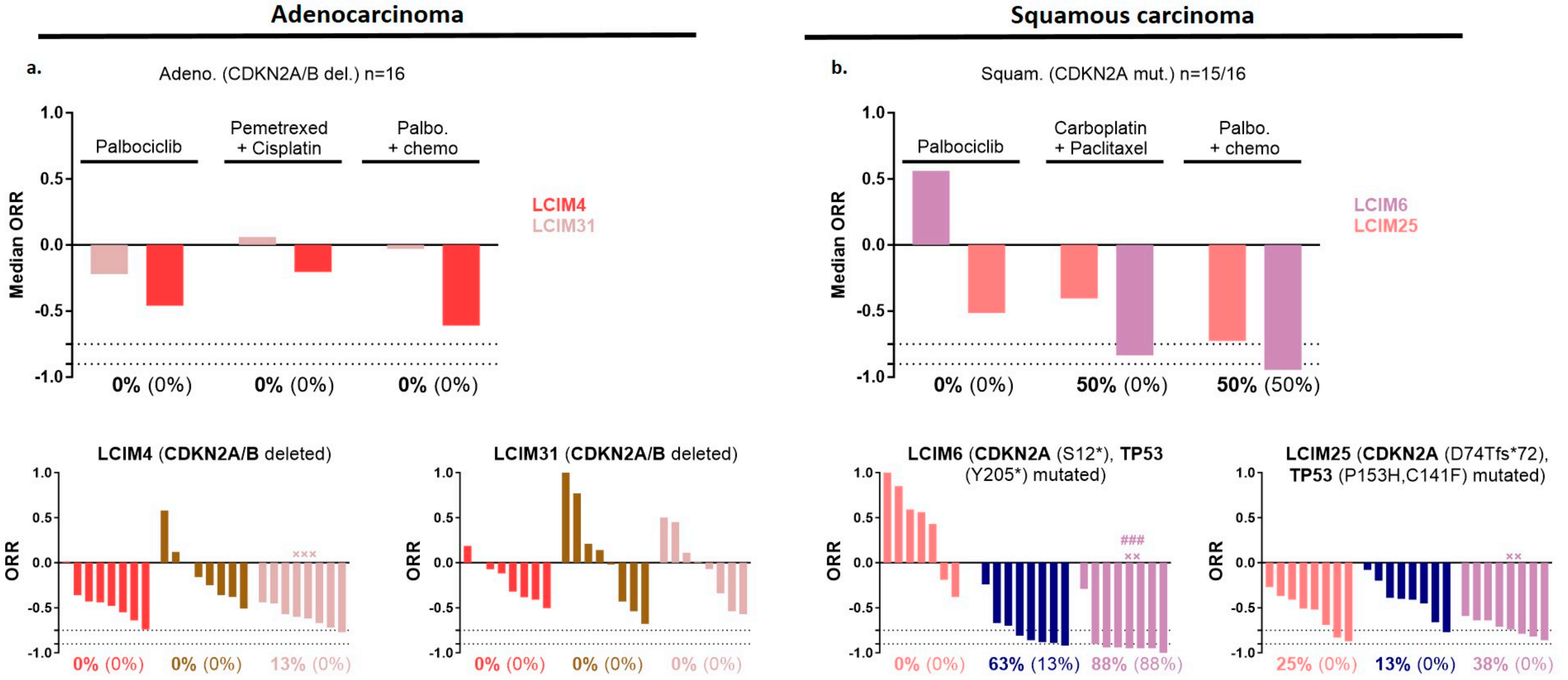

Supplementary Figure S9: *In vivo* efficacy study of MYC targeting and epigenetics targeting in NSCLC PDX models with genomic alterations. a. Histological and genomic characteristics of PDX models with MYC amplification and ARID1A/SMARCA4 mutations. *In vivo* targeting of 2 NSCLC PDXs by birabresib (60 mg/kg, 5x/w, *per os*) (b.) and of 3 NSCLC PDXs by tazemetostat (150 mg/kg, 5x/w, *per os*) (c.). Overall response rate (ORR); the percentage in orange correspond to an ORR lower than −0.75 and the percentage in green correspond to an ORR lower than −0.9. Mean RTV +/- SEM.

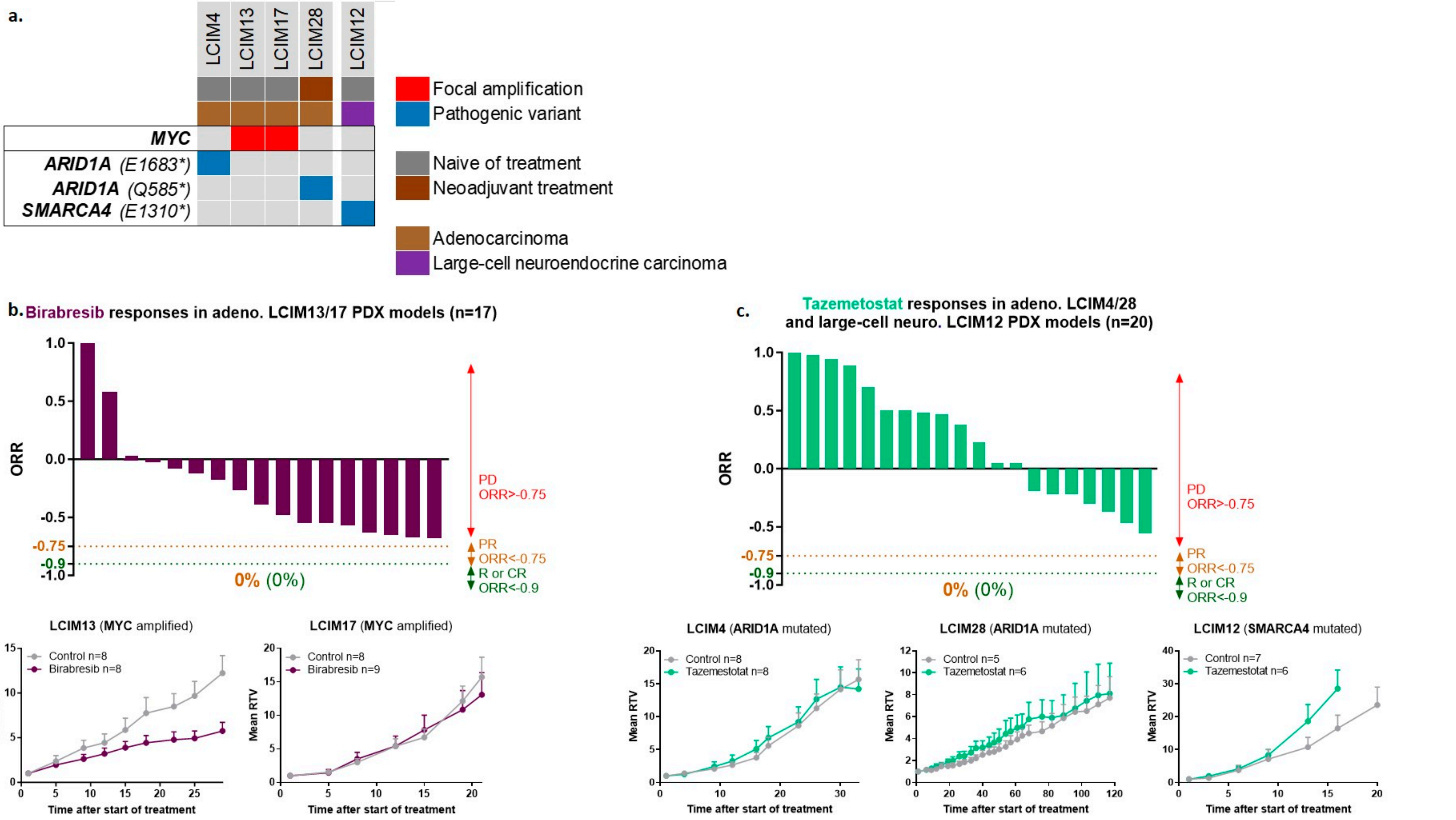

Supplement: Supplementary file 1 [file cancers-16-02785-s001.zip › cancers-3122851-supplementary.pdf]
